# Advancements in Biosensors for Lipid Peroxidation and Antioxidant Protection in Food: A Critical Review

**DOI:** 10.3390/antiox13121484

**Published:** 2024-12-05

**Authors:** Majlinda Daci, Liridon Berisha, Dario Mercatante, Maria Teresa Rodriguez-Estrada, Zongxin Jin, Yeqin Huang, Riccardo Amorati

**Affiliations:** 1 Department of Chemistry, Faculty of Mathematics and Natural Sciences, University of Pristina, Str. Mother Teresa, 10000 Prishtina, Kosovo; majlinda.daci@uni-pr.edu; 2 NanoAlb, Albanian NanoScience and Nanotechnology Unit, Academy of Sciences of Albania, Shëtitorja Murat Toptani, 1000 Tiranë, Albania; 3 Dipartimento di Scienze e Tecnologie Agro-Alimentari, Alma Mater Studiorum-Università di Bologna, Viale G. Fanin 40, 40127 Bologna, Italy; dario.mercatante2@unibo.it (D.M.); maria.rodriguez@unibo.it (M.T.R.-E.); 4 Dipartimento di Chimica “Giacomo Ciamician”, Alma Mater Studiorum-Università di Bologna, Via Gobetti 83, 40129 Bologna, Italy; zongxin.jin2@unibo.it (Z.J.); yeqin.huang2@unibo.it (Y.H.)

**Keywords:** biosensors, peroxidation, antioxidants, enzyme, electrochemistry

## Abstract

This review highlights the progress made in recent years on biosensors aimed at detecting relevant analytes/markers of food peroxidation. Starting from the basic definition of biosensors and the chemical features of peroxidation, here we describe the different approaches that can be used to obtain information about the progress of peroxidation and the efficacy of antioxidants. Aptamers, metal–organic frameworks, nanomaterials, and supported enzymes, in conjunction with electrochemical methods, can provide fast and cost-effective detection of analytes related to peroxidation, like peroxides, aldehydes, and metals. The determination of (poly)phenols concentrations by biosensors, which can be easily obtained by using immobilized enzymes (like laccase), provides an indirect measure of peroxidation. The rationale for developing new biosensors, with a special focus on food applications, is also discussed.

## 1. Introduction

The autoxidation of lipids and proteins under O_2_, reaction, also known as peroxidation, is of significant concern for the food industry as it results in food spoilage, off-flavors (including rancid), and a decline in nutritional value with the loss of nutrients [1]. Antioxidants are of great importance to slow down peroxidation processes, thus allowing increases in food shelf-life and safety and a reduction in food waste. Therefore, the development of quick and affordable methods for tracking the progression of peroxidation and/or the consumption of antioxidants is essential for the advancement of this field. Conventionally, the extent of peroxidation is determined through labor-intensive methods requiring laboratory facilities and/or specialized equipment, such as the determination of peroxides, conjugated dienes, and volatile aldehydes [2]. To further advance research about peroxidation and antioxidants, new techniques that combine user-friendliness, affordability, and chemical significance are needed. In this sense, biosensors are probably the most promising prospect to achieve these goals. As will be described in this review, miniaturized devices can be produced at a relatively reduced cost and could potentially be employed on-field, without specialized equipment or operators. The possibility to attain multi-parameter sensing on a sample of reduced volume would also provide a comprehensive insight into peroxidation, thus potentially allowing a more effective prediction of shelf-life and the effect of antioxidants.

However, because of the chemical complexity and the slow kinetics of peroxidation, the development of suitable biosensors will face problems of selectivity and sensitivity. In this review, we aim at describing the state of the art of biosensor-based methods to measure the progression of peroxidation and the efficacy of the antioxidants. Moreover, we provide a rationale for developing new biosensors, with a special focus on food applications.

## 2. Lipid Peroxidation and Antioxidants

### 2.1. Chemical Mechanism of Lipid Peroxidation

**Scheme 1:**
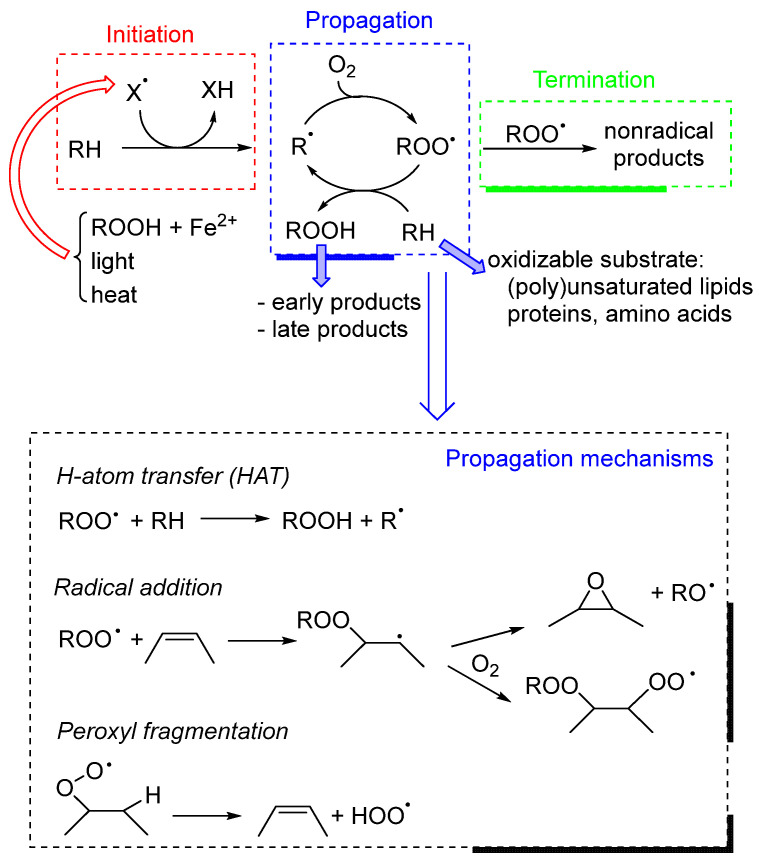
Mechanism of peroxidation.

Lipid-containing foods and emulsions (like mayonnaise) undergo oxidative degradation, which is primarily caused by lipid peroxidation [3,4]. Despite the multiplicity of situations in which peroxidation is observed, the mechanism is similar, consisting of a radical chain reaction carried on by oxygen-centered peroxyl radicals, ROO^•^ [5]. Because of the very fast reaction of carbon-centered (alkyl) radicals R^•^ with (triplet) O_2_, virtually all alkyl radicals are transformed into ROO^•^ even at low O_2_ concentrations, making ROO^•^ the most relevant radical to be considered during lipid peroxidation [6]. Lipid peroxidation can be divided into three broad mechanistic steps, shown in Scheme 1.

1. Initiation consists of the formation of radicals in the systems. In food, this process can be due to various mechanisms: direct reaction of O_2_ with bisallylic C-H groups (usually accelerated by heat) [7], photosensitization [8], and metal-catalyzed decomposition of hydroperoxides [9,10]. It should be remembered that, because of the radical chain nature of peroxidation, several substrate molecules are destroyed by one radical, and even a very small initiation rate is able to produce a dramatic effect over long periods.
2. Propagation is based on the reaction of the (alkyl)peroxyl radical ROO^•^ with the substrate, with the subsequent formation of a hydroperoxide (ROOH) and a carbon-centered radical that reacts with O_2_ forming a new ROO^•^. In lipids, propagation mainly involves polyunsaturated fatty acids (PUFAs), because the bisallylic position is (at room temperature) hundreds of times more reactive toward ROO^•^ than allylic or aliphatic ones [5]. However, the propagation mechanism is in many cases more complex than the one described here; for instance, it may involve ROO^•^ fragmentation to produce HOO^•^ or RO^•^, together with closed-shell oxygenated early products like epoxides and carbonyls [11].
3. Termination is the step in which radicals disappear from the system by reacting with each other (i.e., by bimolecular decay). Typically, two secondary ROO^•^ radicals (like in the case of lipids) generate a carbonyl and an alcohol. While radical–radical reactions are typically fast, the low concentration of radicals nonetheless makes termination a statistically rare event, which may be further slowed down by lipid compartmentalization strategies (i.e., emulsified lipids) [5].

### 2.2. Products

The products of peroxidation can be divided into early products, which are those directly produced during radical chain reactions, and late products, which derive from the degradation or transformation of early products. However, the distinction is not sharp because some products can be formed by both mechanisms.

#### 2.2.1. Early Products

**Scheme 2:**
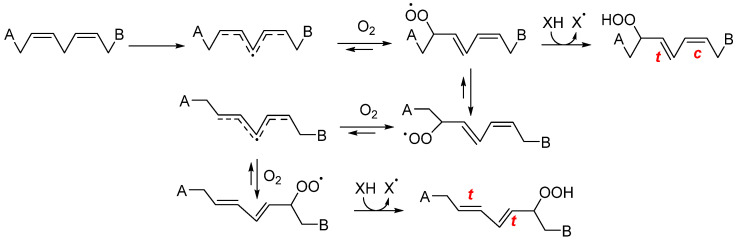
Simplified mechanism of the formation of early oxidation products from linoleate residues. A and B represent the two ends of the fatty acid, and XH is a generic H-atom donor. From Ref. [6].

Early products are represented mainly by hydroperoxides, especially in the case of PUFA. In the case of linoleate residues, for example, the complete product distribution has been clarified as reported in Scheme 2, showing that the relative position of the double bonds and their *cis/trans* isomers depend on how fast the peroxyl radicals are quenched by H-atom donors [12]. Therefore, during the peroxidation of triacylglycerols, *trans*–*trans* hydroperoxides are typically formed in much larger amounts than *cis*–*trans* hydroperoxides. However, their relative concentration is reversed in the presence of antioxidants [12]. Polyunsaturated lipids form hydroperoxides having conjugated double bonds, which can be easily detected spectrophotometrically [13].

The product distribution becomes more complex with an increasing number of double bonds in the fatty acids, due to the occurrence of fast cyclization reactions that may involve both peroxyl and alkyl radicals [12]. In the case of substrates having only one unsaturation, like oleic acid and cholesterol, the H-atom abstraction by ROO^•^ that would yield hydroperoxides is not thermodynamically favored; thus ROO^•^ addition to the double bond also takes place, in a competitive manner. Radical addition on non-conjugated double bonds typically generates an epoxide and a secondary alkoxyl radical, RO^•^ (see Scheme 1) [12], which can rearrange to an alpha-hydroxyl alkyl radical that, upon reaction with O_2_, finally yields a ketone and HOO^•^ [14].

#### 2.2.2. Late Products

The formation of late products is kindled by the homolytic or heterolytic reaction of unstable hydroperoxides. For this reason, the hydroperoxide level increases only at the beginning of lipid peroxidation, followed by a decrease as they convert into more stable secondary products that tend to accumulate [15]. In fact, it is common practice to evaluate the oxidative stability of lipid materials by measuring the content of both hydroperoxides and late products (typically volatile and non-volatile aldehydes) [16].

**Scheme 3:**
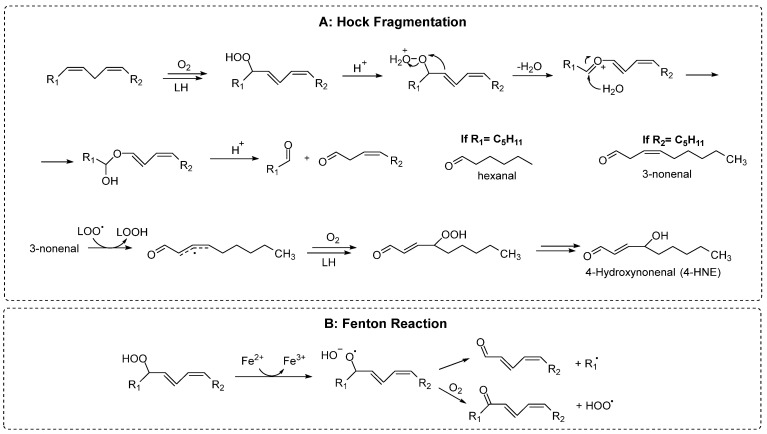
Examples of mechanisms leading to the formation of late products of lipid peroxidation.

Although the reaction network leading to secondary products is very complex (for a comprehensive review, see Refs. [5,6,12]), two main pathways of hydroperoxide decay can be recognized: Hock fragmentation and radical homolysis. The latter is catalyzed by reduced transition metals (like Fe^2+^) and provides alkoxyl radicals, which then may rearrange into alpha-hydroxy alkyls, or fragment into alkyl radicals and aldehydes (see Scheme 3). This reaction is of paramount importance in the initiation of peroxidation reaction, because traces of metals and hydroperoxides are always present in lipid materials [17].

Hock fragmentation is instead catalyzed by acids and heat, and through a non-radical pathway transforms a hydroperoxide into two aldehydes [5]. It was recently shown that this mechanism is fundamental for forming oxylipins of cholesterol [18]. The nature of the aldehydes formed during lipid peroxidation critically depends upon the kind of fatty acid being oxidized. As expected, omega-3 lipids provide aldehydes with a shorter chain (and higher volatility) than omega-6 ones [19]. Hydroperoxides can also react with alkenes forming epoxides, which in turn can be attacked by water or acids forming diols or diesters, respectively [20]. In addition to carbonyls and epoxides, other minor products that derive from the advanced oxidation of aldehydes are also formed, like furans, aldol condensation products [19], and short-chain acids like acetic and formic acid [15], which are the main water-soluble volatiles that are detected by the Rancimat assay [21].

Late products may be divided into two categories: volatile and non-volatile. The former are the ones that give lipid peroxidation its rancid, off-flavor. As they derive from the fragmentation of the fatty acid carbon chain, non-volatile products are most often represented by the fragment of the carboxylic acid, while volatile products are from the distal part [3].

## 3. Antioxidants

**Scheme 4:**
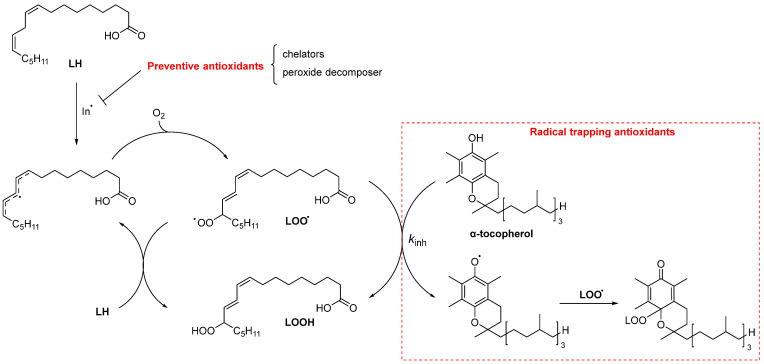
Mechanism of action of antioxidants.

In the context of lipid peroxidation, antioxidants are natural compounds or synthetic additives able to slow down the formation of oxidation products. They can be divided into preventive and chain-breaking antioxidants, depending on whether they antagonize the initiation or propagation step, respectively [22]. Preventive antioxidants are represented, for example, by metal chelators (like phytic acid) [23] which inactivate or remove iron ions from the water/lipid interface [24]. Chain-breaking (or radical-trapping) antioxidants act by directly trapping ROO^•^ [25] or by catalytic HOO^•^/ROO^•^ quenching (see Scheme 4) [26].

Most small-molecule natural antioxidants belong to the chain-breaking class, including tocopherols, phenolic acids, flavonoids, etc., as well as synthetic additives like *tert*-butylhydroquinone (TBHQ), butylated hydroxytoluene (BHT), and propyl gallate (PG) [25]. All these antioxidants are consumed during their activity, and for this reason, they provide inhibition for a limited time, which depends on their concentration [7].

Most natural lipids contain antioxidants. Seed oils, for example, have relatively large amounts of tocopherols or tocotrienols, while olive oil also contains polyphenols like oleuropein and hydroxytyrosol [27,28]. As chain-breaking antioxidants are consumed during peroxidation, the determination of their level can represent an indirect way to measure the extent of radical attack [29,30].

**Figure 1:**
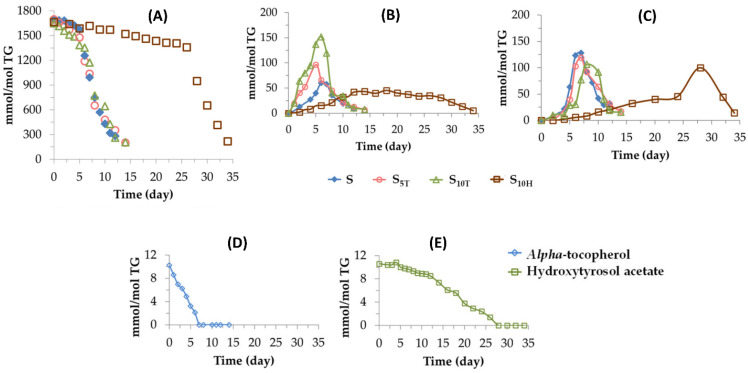
Concentration of (**A**) bisallylic groups; (**B**) *c,t* hydroperoxides; (**C**) *t,t* hydroperoxides; (**D**,**E**) antioxidants during the autoxidation of sunflower oil at 70 °C under different conditions: without added antioxidants (S), with 5 mM α-TOH (S5t), with 10 mM α-TOH (S10T), or with 10 mM hydroxytyrosol acetate (S10H). Figure adapted from Ref. [15].

Figure 1 reports the variation in the concentrations of the oxidizable lipids of *t*,*c* and *t*,*t* hydroperoxides and of α-tocopherol (α-TOH) or hydroxytyrosol acetate during the peroxidation of sunflower oil at 70 °C [15]. These results show that at the beginning of the reaction, there is a slow consumption of the bisallylic groups, because of the presence of the antioxidants. However, under these conditions, hydroxytyrosol acetate was demonstrated to be a much better antioxidant than α-TOH (see Figure 1B,C), because it resulted in a longer inhibition period and a stronger reduction in hydroperoxide production. In Figure 1D,E, it is shown that α-TOH is consumed faster than hydroxytyrosol acetate, suggesting that the disappearance of antioxidants represents an index to describe the oxidative stability of the system.

## 4. Biosensors for Lipid Peroxidation Analysis

### 4.1. What Are Biosensors

**Figure 2:**
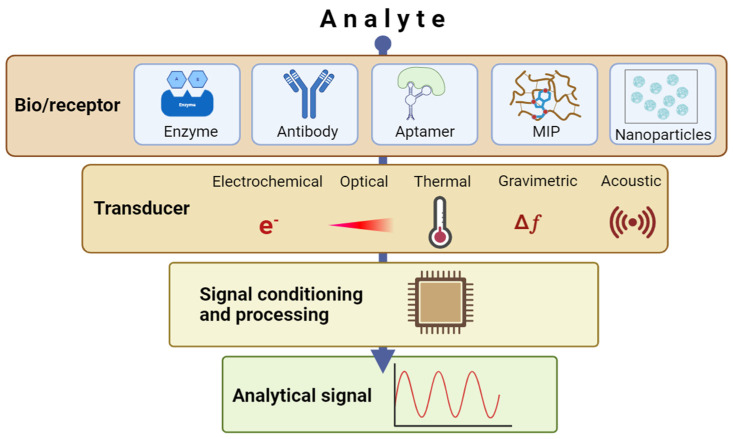
General scheme of biosensor operation principle.

According to the IUPAC, biosensors are defined as “a device that uses specific biochemical reactions mediated by isolated enzymes, immunosystems, tissues, organelles, or whole cells to detect a chemical compound” [31]. The sensors are constituted by a recognition part, which enables the production of the analytical signal through the interaction with the analyte or group of analytes to which it is specific or selective. This interaction between the receptor and the analyte/s produces a change in a physical parameter, which can be electrochemical, optical, thermal, electronic, gravimetric, or acoustic, and transmitted through the transducer to the electrical circuit (amplifier), thus giving a signal that is proportional to the concentration of the analyte [32]. The selectivity of the sensor depends on the receptor chosen for its construction. If the recognition part is a biological receptor, they are biosensors. Based on the type of bioreceptor, they are classified into enzymatic biosensors, immunosensors, aptasensors, cell sensors, DNA sensors, etc., as summarized in Figure 2.

#### 4.1.1. Enzymatic Biosensors

The ability of enzymes to act as specific biocatalysts has largely been utilized for the development of biosensors that employ a variety of transducers, including thermal, optical, and electrochemical ones. The enzyme glucose oxidase, which is present in the biosensor for glucose, catalyzes the conversion of glucose to gluconic acid and, when exposed to oxygen, oxidizes to produce hydrogen peroxide. During such an enzymatic reaction, all the changes that occur can be used to produce an analytical signal proportional to the glucose concentration. For instance, the production of gluconic acid leads to a change in pH that is proportional to the amount of glucose present in the solution, so by fixing the enzyme on the surface of the pH electrode, a biosensor for glucose can be built.

The oxygen used for the reoxidation of the enzyme also enables the determination of glucose, because for every molecule of glucose that is oxidized, one molecule of oxygen is consumed. Therefore, due to the equivalence with the amount of oxygen, the quantitative determination of glucose can be carried out by means of an oxygen electrode [33]. Moreover, the determination of hydrogen peroxide as a product of enzyme reoxidation makes it possible to determine glucose, because the amount of peroxide generated on the surface of the electrode is equivalent to the amount of glucose [34].

The use of enzymes as bioreceptors represents a good opportunity to build very specific biosensors, and many research groups over the years have investigated the construction of enzymatic biosensors with a focus on their application in different types of samples, such as biological fluids, food, water, environment, etc. [35,36,37,38]. The challenge of developing specific biosensors to be applied to real complex samples has been faced by using modifiers in transducers that enable operation at lower potentials; this approach facilitates electron transfer and thus reduces the possibility of oxidation/reducing interferences at lower operating potentials [39,40,41,42]. Research on electrochemical biosensors has gone even further, by trying to make a direct connection between the active center of the enzyme with the electrode through nanowires and nanoparticles, thus enabling the direct transfer of electrons between the electrode and the enzyme; this results in an electrical signal with higher sensitivity [43,44,45].

The new nanomaterials that imitate the catalytic activity of enzymes are called nanozymes, which have many advantages over enzymes, such as high stability, high resistance to harsh environments, low cost, the possibility of mass production, and a longer lifespan [46]. By exploiting this concept, nanostructures built on the surface of the sensor able to mimic the catalytic effect of enzymes have been developed [47,48,49].

#### 4.1.2. Aptasensors

Aptamers are synthetic oligonucleotides that exhibit the ability to bind a particular target molecule, functioning akin to “chemical antibodies” and facilitating the creation of specific biosensors. Aptamers are obtained through the Systematic Evolution of Ligands by Exponential Enrichment (SELEX) process [46,50] where, in the presence of a target molecule, the ligands library of oligonucleotides with high affinity to the analyte are cyclically partitioned and reproduced, thus increasing the number of aptamers with selectivity to the analyte. These aptamers can be used with various transducers, providing analytical signals with high specificity and application in food control [51,52,53], health [54,55,56], environment, etc. [57,58,59,60].

#### 4.1.3. Immunosensors

The use of antigen–antibody interaction (lock and key) for the detection of low concentrations with high selectivity by using antibodies as bioreceptors has aroused great research interest in the field of biosensors [61]. The fixation of antibodies on the surface of the biosensor produces an analytical signal in the presence of the antigen that is proportional to its concentration. Depending on the detection method, immunosensors can be labeled with electroactive species [62] or can be label-free [63]. Labeled antibodies are used to increase sensitivity and specificity for applications in complex sample matrices. To amplify the signal, immunosensors incorporating a second antibody, linked to an electroactive compound, have been developed. After binding to the antigen attached to the first antibody, it enables the generation of an electrochemical signal. Enzyme-labeled antibodies amplify the signal in the presence of the enzyme substrate, which can be electrochemically detected. The modification of second antibodies with metal nanoparticles is a good possibility for signal amplification in electrochemical sensors; this indeed enables the determination of the metal, which is proportional to the concentration of the antigen to which the second antibody is bound [62,64,65,66].

#### 4.1.4. Molecularly Imprinted Polymers (MIP)

They are also known as “plastic receptors”, as they are built through a polymerization reaction in the presence of the analyte. After the polymerization process, the analyte is removed from these polymers with solvent, leaving vacant spaces and creating a suitable structure for a selective binding site for the analyte. This type of receptor has found great application in the construction of sensors, enabling the generation of the analytical signal in the presence of the analyte with minimal interference. In conjunction with an electrochemical interface, the use of MIP has shown an application in complex samples, thus enabling the production of sensors with low cost and high stability compared to biosensors, which require more specific storage conditions and have higher costs. MIP sensors have been built to detect toxins in food [67], biomedical analysis [68], food allergens [69], food adulterants [70], etc. However, the signal produced by the connection between the polymer and the analyte often does not reach a high analytical sensitivity. Attempts have been made to incorporate various nanomaterials [68,69,71,72] that facilitate the signal transmission process in the production of the electrical signal, thus making possible the determination of analytes at low concentration levels in quite complex samples [70,73].

### 4.2. Antioxidant Analysis

Over the past decade, the utilization of antioxidants in the food industry has significantly increased. They are frequently used to ensure the quality of food products and prevent their oxidation [1,74]. The most widely used natural antioxidants in food are ascorbate (vitamin C), tocopherols (vitamin E), and polyphenols (including flavonoids and phenolic acids) [75].

Among synthetic antioxidants, the most commonly employed in food formulations are butylated hydroxyanisole (BHA), BHT, PG, and TBHQ [76,77].

As discussed in Section 3, as antioxidants are consumed during peroxidation, antioxidant levels can also provide a measure of the overall oxidation stability of the material. Conventional techniques like gas chromatography (GC) [78,79] and liquid chromatography (LC) [78,80] are costly, time-consuming, and involve intricate sample preparations. Colorimetry methods, on the other hand, although much simpler than GC and LC, are also time-consuming and require larger amounts of samples and reagents [81], in addition to their frequent lack of specificity which can lead to quantification problems in complex food matrices. To overcome these limitations, various sensors and biosensors have emerged as valid tools for assessing antioxidant levels in food. These devices effectively identify and measure specific analytes within food matrices, offering high sensitivity, specificity, fast analysis performance, and minimal sample pre-treatment requirements, coupled with the convenience of miniaturization [82,83,84,85,86,87,88].

#### 4.2.1. Electrochemical Biosensors for Polyphenol Detection

The evaluation of the quantity of phenols in a sample (food products, beverages, plant extracts, or environmental samples) can be performed by measuring the concentration of specific phenolic compounds or by determining the total phenolic content (TPC). Given that about 9000 plant phenolic compounds have been identified to date, this technique faces a challenging task [89]. Alternatively, the TPC approach is less time-consuming, involving the semiquantitative evaluation of complicated multicomponent samples (plant-based materials, processed foods, or environmental matrices). In a thorough examination of biosensors tailored for studying rosmarinic acid, David et al. highlighted persistent challenges within this field [90]. One critical challenge lies in enhancing selectivity towards closely related compounds, such as other hydroxycinnamic acids. To face this challenge, it is necessary to utilize chemically modified electrodes, including techniques like molecularly imprinted polymers. These modifications have the potential to enhance selectivity while simultaneously lowering detection limits. By doing so, they have the potential to improve the accuracy and effectiveness of electroanalysis for rosmarinic acid [91].

#### 4.2.2. Enzyme-Based Biosensors

**Figure 3:**
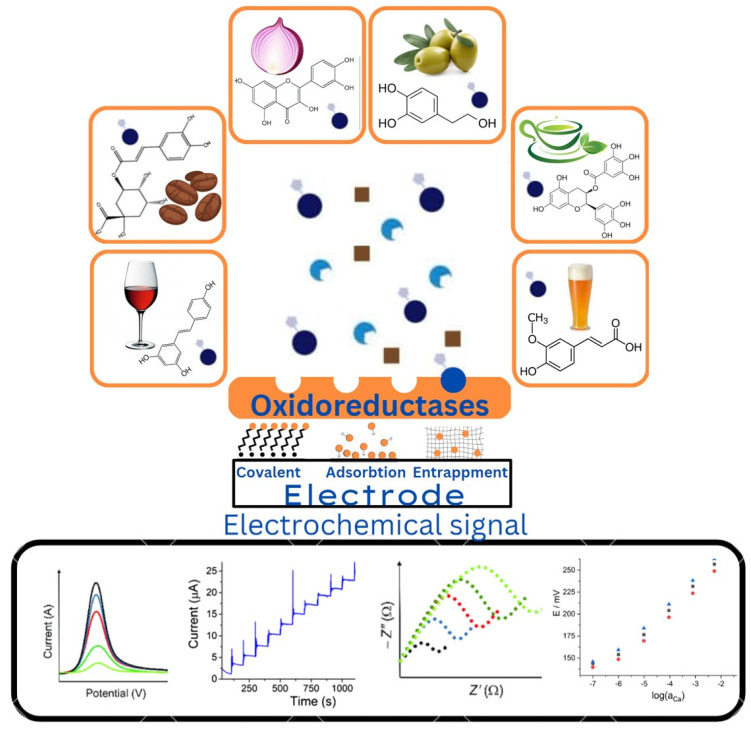
Oxidoreductase used in biosensors for the quantification of natural phenolic compounds.

In biosensors, the most used enzymes for the detection of antioxidants in food belong to the oxidoreductase, hydrolase, or lyase categories (Figure 3). These enzymes can identify particular substrates and produce quantifiable signals, which enable biosensors to precisely determine the amount of antioxidants present in dietary samples. The relationship between how much inhibitor is present and how much it slows down or reduces the enzyme’s activity enables both quantitative and qualitative assessments of analytes [82,83,84,85,86,87,88].

Oxidoreductase enzymes facilitate the transfer of electrons from the phenolic substrate to an electron acceptor, usually molecular oxygen. Once they are oxidized by the enzyme, phenolic compounds are converted into quinone derivatives; the latter undergo reduction at the electrode surface of the biosensor back into the initial phenolic compounds, leading to the generation of electrochemical signals. The electrochemical signal generated during this process is proportional to the concentration of phenolic compounds present in the sample [85,86,87]. The application of biosensors based on phenol oxidase for the identification of antioxidants in food is a viable strategy with several benefits. Enzymes including tyrosinase and laccase belong to this group [85,87].

#### 4.2.3. Tyrosinase-Based Biosensors

**Scheme 5:**
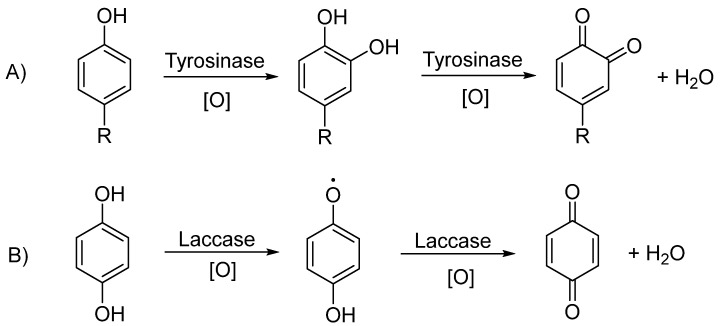
Mechanism of enzymatic oxidation of phenols and polyphenols by tyrosinase (**A**) or laccase (**B**).

Tyrosinase is a copper-containing enzyme found in plants, animals, and fungi that catalyzes the hydroxylation of mono-phenols to *ortho*-diphenols via the monophenolase reaction and the oxidization of *ortho*-diphenols to *ortho*-quinone through the diphenolase reaction (see Scheme 5A). L-tyrosine participates as a substrate in both reactions. In tyrosinase-based biosensors, the reversible electrochemical reduction of *ortho*-quinone is measured. The obtained signals reflect the concentration of polyphenols in the solution.

For detecting phenolic antioxidants in beer, a tyrosinase-based electrochemical biosensor showed a good correlation with the Folin–Ciocalteu (FC) method, even though the values obtained from the FC assay were significantly higher [91]. This indicates the selective nature of the biosensing platform as the application of tyrosinase ensures that the electrochemical signal is acquired only from the phenolic substrates of the enzyme, thus eliminating any interference and limiting phenol overestimation.

A nanostructured surface tyrosinase biosensor named poly-(3,4-ethylenedioxythiophene)-tyrosinase/sonogel-carbon (PEDOT-Tyr/SNGC) was used for evaluating the polyphenol content of beers and wines. Using caffeic acid as the reference polyphenol, the biosensor exhibited a high sensitivity with a limit of detection of 4.3 µM and a correlation coefficient (R^2^) of 0.9987. The advantages of using this biosensor lie on its simplicity and rapidity, with enzyme and polymer deposition occurring simultaneously in one step for a few minutes while delivering an instantaneous low-cost response [92].

#### 4.2.4. Laccase-Based Biosensors

Similar to tyrosinase, laccase is an oxidoreductase enzyme that catalyzes the oxidation of a wide range of phenolic and non-phenolic compounds. Laccase catalyzes the oxidation of substrates by transferring electrons from the substrate to molecular oxygen, resulting in the formation of water as a byproduct. During this process, laccase generates reactive oxygen species, such as hydroxyl radicals, which can further oxidize substrates (Scheme 5B) [87,93].

Boubezari et al. created a new biosensor using laccase encapsulated within a chitosan–galactomannan composite to identify the phenolic chemicals in olive oil [94]. Pyrocatechol was successfully identified by the biosensor, and the measurement of *ortho*-quinone at a potential of −0.447 V served as the basis for the detection. In the concentration range from 10^−16^ M to 10^−4^ M, the biosensor demonstrated a record-low LOD (limit of detection), and its voltammetric response stayed constant for more than two weeks. Moreover, the chitosan–galactomannan composite did not lose its laccase activity [94].

Salamanca-Neto et al. [95] presented a novel approach to measure the chlorogenic acid content in brewed coffee beverages, by using a biosensing device fabricated with platinum nanoparticles and laccase. The authors found that, under optimized experimental conditions, the biosensing device showed a linear response for chlorogenic acid in the range of 0.56–7.3 µmol L^−1^ with high accuracy and sensitivity. They also found that the biosensing device had a storage stability of 30 days and a lifetime of 150 measurements, indicating its potential for practical use in analyzing the chlorogenic acid content in brewed coffee beverages. The detection and quantification limits of the biosensor were found to be 0.18 and 0.59 µM, respectively, which are comparable to those of traditional analytical methods, such as HPLC [95].

Mohtar et al. [37] presented a novel biosensor utilizing laccase for the detection of polyphenols in propolis. The biosensing scheme is based on a nanocomposite film of laccase immobilized on gold nanoparticles electrodeposited on a screen-printed carbon electrode modified with polypyrrole (Ppy) through in situ electropolymerization (Ppy/Lac/AuNPs/SPCE). Polyphenols contained in ethanolic extracts of propolis were first oxidized by laccase, then reduced by Ppy/Lac/AuNPs/SPCE providing an amperometric response. The results showed a linear response in the concentration range from 1 to 250 μM expressed as caffeic acid, with a limit of detection of 0.83 μM. The biosensor exhibited a rapid analysis time (15 min), good selectivity, stability, and reproducibility [37].

To identify hydroquinone and other phenolic chemicals in wine and blueberry syrup, another laccase-based biosensor was immobilized onto a screen-printed carbon electrode enhanced by gold nanoparticles and graphene nanoplatelets (LACC/AuNP/GNPl/SPCE). A low detection limit of 1.5 µM was obtained, with a linear HQ concentration range from 4 to 130 µM. The biosensor demonstrated good repeatability and reproducibility; however, additional validation is required to determine its selectivity for phenolic chemicals in comparison to other interfering molecules [96].

In a recent study by Mediavilla et al. [97], the total polyphenol content was determined in rice leaf extracts, wine, and juice, by using a direct fluorescence assay. A bioconjugate, generated by the laccase enzyme (from Trametes versicolor) and carbon nanodots, was biofunctionalized and used as a fluorescent label for the new biosensor. The biosensor detected phenolic chemicals (such as gallic acid, GA) by the enzymatic reaction of the bioconjugate and GA, generating quinones that led to quenched fluorescence. The quantification and detection limits were 25 μM and 7.4 μM, respectively [97].

Additionally, Gomes et al. [98] conducted a study wherein they immobilized laccase from *B. rhodina* MAMB-05 on carboxymethyl-botryosphaeran to measure the presence of quercetin in drugs, drinks, and biological materials. In the range of 4.98–50.0 × 10^−8^ mol L^−1^, the biosensor displayed a linear response for quercetin, with a predicted detection limit of 2.6 × 10^−8^ mol L^−1^ [98].

To identify a wider range of phenolic chemicals, enzymatic biosensors containing both tyrosinase and laccase enzymes have been developed [99,100]. To detect catechol in wines, Apetrei et al. [99] looked into the self-encapsulation of a polyphenol oxidase enzyme extract (synthesized by *T. pubescens*) within a conducting polypyrrole material. The three biosensor types that were compared were Chem-Ppy-TP (chemically synthesized Ppy with enzyme extract), Bio-Ppy (biosynthesized Ppy-based biosensor), and Bio-Ppy-TP (Bio-Ppy with additional enzyme extract). All three biosensors effectively identified catechols in fruit beverages (such as pomegranate, blackberry, and blueberry) at low concentrations (1–60 μM) [99].

#### 4.2.5. Metal–Organic Framework

The use of metal–organic frameworks (MOFs) as catalysts [101] in the construction of sensors has also aroused great interest. This is primarily because of the porous nature of the crystalline material and the large active surface area [102]. The use of MOFs in the construction of electrochemical sensors is possible by mixing them with other materials that enable electrochemical signal transduction. The determination of rutine is a typical example based on MOFs, using cerium (Ce) as an ion with catalytic properties and poly(3,4-ethylenedioxythiophene (PEDOT) as a good conductor to build a Ce-PEDOT nanocomposite with catalytic properties [103].

Carbon nanofibers, synthesized starting from cubic nanoparticles of glycosyl-MOF-5 and a suspension of polyacrylonitrile through electrospinning, have provided a good sensitivity in the electrochemical determination of quercetin using a glassy carbon electrode [104]. Another ultrasensitive sensor for the determination of baicalin was built from graphitized carbon nitride, single-walled carbon nanotubes, and reduced graphene oxide by the electrodeposition of cyclodextrin-MOF and was successfully applied to real samples (human serum and bear bile scutellaria eye drops) [105]. The use of mesoporous carbon mixed with a zirconium fumarate metal oxide framework increased the conductivity, specific surface area, and pore volume and showed an electrocatalytic effect in the determination of gallic acid and luteolin in green tea and urine samples [106]. Using MOF as a nanocomposite in the construction of electrochemical sensors reduced the oxidation overpotential and increased the sensitivity for flavonoids and phenolic acids, such as kaempferol [107,108], catechin and sunset yellow [109,110], caffeic acid [111,112], catechol [113] and phenolic acids [114], gallic acid [115], coumaric acid [116], etc.

Electrochemical sensors with only MOF have also been built for the determination of ascorbic acid and L-tryptophan [117,118]. However, the application of MOF in the development of the sensors also has shortcomings, such as low selectivity for specific compounds, costly preparation, and the use of non-green chemicals. In any case, the preparation of sensors with MOFs is a good opportunity for the preparation of Point-Of-Care (POC) sensors, since they shorten the analysis time and enable on-time quality control for several widely consumed food products.

The use of enzymes as receptors of biosensors shows their limitations due to their low durability and stability, expensive mass production, and the limited environments where they can be employed; all these aspects have led researchers to build new materials that imitate their catalytic activity.

#### 4.2.6. Nanozymes

Nanozymes are nanomaterials that have enabled the replacement of enzymes as bioreceptors in the construction of biosensors, especially peroxidases, catalase, and superoxide dismutase (Table 1).

Nanozymes built with phosphorus-doped carbon spheres having a nanoporous structure were used in the voltammetric determination of baicalin and luteolin [119]. The electrochemical determination of phenolic compounds has been successfully carried out by constructing a nanocomposite with enzyme-like properties from manganese oxide (IV) and quantum graphene nanodots, which has also proven its performance in the colorimetric determination of catechol and dopamine [120]. The increase in the selectivity of nanozymes is achieved with the construction of single-atom nanozymes (SANs), managing to be anchored in the metal oxide framework in porous carbon; this enables the simultaneous electrochemical determination of catechol and hydroquinone [121].

#### 4.2.7. Molecular Imprinted Polymers (MIPs)

A MIP made of methacrylic acid and ethylene glycol dimethacrylate as a cross-linking agent, in the presence of α-tocopherol as a template, was able to bind α-tocopherol in edible oils with a better affinity than TBHQ and Trolox (the hydrosoluble α-tocopherol analog). The binding extent was measured by Raman spectrometry [122]. Despite these interesting results, no further attempts have been made to assess the capability of this method in food samples undergoing oxidation.

### 4.3. Peroxidation Analysis

#### 4.3.1. Hydroperoxides

Since hydroperoxides are the primary early peroxidation products, determining their presence is crucial to understanding how food oxidizes. Regretfully, research has mostly focused on hydrogen peroxide, with little attention paid to determining alkyl hydroperoxides (the primary products of peroxidation), while H_2_O_2_ is merely a side product (see Scheme 1). The only example of alkyl hydroperoxide detection is represented by an electrode featuring horseradish peroxidase absorbed on polypyrrole with amperometric detection. This method was able to quantify hydroperoxides like *tert*-butylhydroperoxide in a mixed aqueous–organic system [123]. Many biosensors have been developed for the determination of hydrogen peroxide, enabling the monitoring of food quality. Using nanomaterials for the construction of biosensors increases sensitivity by providing an easier connection and electron transfer between the receptor and the transducer.

For instance, cress peroxidase entrapped on ferrimagnetic nanoparticles with amperometric detection [124], organic–inorganic hybrids made of peroxidase–copper phosphate [125], and AgNPs coupled with ferrous disulfide as peroxidase mimics [126] can be considered attempts to build, through nanomaterials, chemical receptors for H_2_O_2_ that mimic the function of enzymes (also called nanozymes).

The use of single-atom catalysts has aroused great interest in various types of electrochemical energy conversion/storage systems, including oxygen reduction, oxygen evolution reaction, hydrogen evolution reaction, and CO_2_ reduction [127]. MOFs represent a promising approach to building sensors for measuring hydrogen peroxide, possibly replacing precious metals and enzymes [128]. Different modification elements have been used for hydrogen peroxide detection, such as cobalt-MOFs [129], trimetallic-MOF nanosheets [130], magnetic covalent organic frameworks [131], Au@Pt nanoflower MOFs [128], metal–organic Langmuir–Blodgett films as electrocatalysts [132], and metal nanoparticle/MOF nanozymes [133] (see Table 2).

#### 4.3.2. Aldehydes

Specific detection of peroxidation-derived aldehydes by using biosensors has not yet been developed. However, 5-hydroxymethylfurfural (HMF) can be considered an example of how to detect aldehydes, despite not being a direct product of peroxidation. HMF originates from the reaction of reducing sugars and proteins during heating through the Maillard reaction, and it is known for its potential adverse effects on human health. The determination of HMF is an important parameter used as an indicator of the quality of food products and the way they have been processed and stored. The construction of a sensor with high specificity for the determination of HMF is a challenge that has been addressed in different ways (see Table 3). It has also been proposed to determine the “furanic index” through which it is claimed to determine food freshness, all furanic derivatives thus being determined as products of the Maillard reaction [134] despite the fact that furans can also be generated by oxidation.

Different types of MIPs have been employed in electrochemical sensors for the determination of HMF and its derivatives, such as a low sensor for 2-furaldehyde detection in beverages using in situ polymerization [135] and electropolymerized molecularly imprinted polymers combined with salting-out assisted liquid–liquid extraction (SALLE)) for the detection of HMF [136]. Electrochemical sensors have been developed for the determination of HMF in honey, coffee, and date molasses, using electrode modifiers such as nickel oxide nanoparticles [137], Cu-Ni bimetallic microparticles on copper electrodes [138], NiO/carbon black [139], Ag nanoparticles incorporated onto carboxymethyl cellulose [140], silver microdendrites [141], and the electrosynthesis of Co-based layered double hydroxides [142]. Graphene quantum dots incorporated in a NiAl_2_O_4_ nanocomposite based on MIP showed increased sensitivity for HMF and were successfully applied in HMF determination in coffee samples [143].

#### 4.3.3. Volatile Aldehydes (VA) and Protein Carbonyls (PC)

Different sensors have been built for the determination of volatile aldehydes using different methods and techniques to improve selectivity [144]. Sensors for nonanal determination incorporate Au nanoparticles to increase the MIP conductivity, transferring the resistance change as signal for the analyte [145]. Hian et al. [146] enhanced the selectivity for gas sensing using MOF, with zinc oxide and zeolitic imidazole framework as a molecular sieve for the detection of formaldehyde. The 3D structure of a Co_3_O_4_-derived MOF was also successfully applied to determine the formation of formaldehyde, due to its higher porosity and presence of active sites [147]. The use of imprinted polymers as receptors and their modification with nanomaterials (carbon nanomaterials) that change their electroactivity in the presence of volatile aldehydes is a good possibility for building resistive sensors that can determine this group of biomarkers.

The determination of protein carbonyls is very important as a biomarker of oxidation in protein-containing food. One of the methods used to determine protein carbonyls is the detection of the dinitrophenol hydrazone (DNP)-carbonyl product from the reaction of dinitrophenylhydrazine (DNPH) with the carbonyl groups in proteins. Protein carbonyls can be determined by using high-specificity antibodies against DNP or by measuring absorbance. Entrapment of DNPH at the electrode surface using Nafion was successfully applied to the electrochemical determination of protein carbonyls [148].

#### 4.3.4. Metals

Iron or copper ions are often involved in the peroxidation of organic materials and food as catalysts for radical production through the Fenton reaction. Therefore, their presence in excess represents an important initiating factor that greatly increases the peroxidation of the material. The Fe or Cu ion recognition is similar to that of heavy metals (cadmium (Cd), lead (Pb), arsenic (As), mercury (Hg), and zinc (Zn)), which are contaminants that can be found in food. Due to their negative effects on the human organism, their fast determination at low concentration levels is of paramount importance. The development of electrochemical sensors for the simultaneous determination of heavy metals has aroused great interest in many research groups.

The most used techniques are the voltammetric techniques, which enable the simultaneous determination of heavy metals; increased sensitivity and lower detection limits (LOD) have been achieved by using different modifiers, as summarized in Table 4. The presence of lead and cadmium was determined in rice and water using carbon nanomaterials (like MWCNT) as modifiers of the electrode surface, obtaining an LOD of 2.7 nM and 0.9 nM for Pb and Cd, respectively [149]. Electrochemically reduced graphene oxide on bismuth film electrodes has successfully been applied for the determination of Pb and Cd in milk samples [150].

The electrochemical sensor for Cu at low concentrations has been built based on the electrocatalytic effect of Cu in the hydrogen evolution reaction, which gives a signal that is proportional to the accumulated amount of Cu on the surface of the pencil graphite electrode modified with a strong ligand chelator (*p*-aminobenzyl-C-functionalized cyclam) for Cu^2+^ [151]. This method has lowered the detection limit for Cu to 1.1 nM.

The simultaneous determination of heavy metal ions based on the construction of graphene aerogel nanocomposites increases the conductivity and also facilitates the transfer of electrons through the metal oxide framework (MOF-UiO-66-NH_2_) [152], which serves as accumulation points for heavy metals due to the interaction of hydrophilic groups with metal ions. Through this operating principle of the sensor, it has been possible to simultaneously determine Cd^2+^, Pb^2+^, Cu^2+^, and Hg^2+^ with very high sensitivity and selectivity in water samples.

Regarding iron, an ion-selective electrode based on polyaniline, a conducting polymer, and chelating agents allowed for the potentiometric detection of Fe^2+^ or Fe^3+^ ions in aqueous solution containing albumin [164]. Ferric ions could be monitored in wine by a biosensor based on a field effect transistor, where a network of carbon nanotubes act as the conductor channel and transferrin as the Fe^3+^ binding receptor [123].

#### 4.3.5. Polar Compounds

Deep frying is one of the most popular food-cooking processes, due to the sensory traits that it confers to food. However, repeated deep frying promotes different chemical reactions, including lipid hydrolysis, oxidation, and polymerization [165].

The degradation products released during deep frying cause changes in the dielectric constant of the frying oil, which is related to their level of total polar compounds (TPCs), the only legally recognized parameter to evaluate the overall frying oil degradation. A high content of TPCs in the frying oil increases the dielectric moment, which can be used to monitor the oil quality [166].

Due to the toxicity of some of the produced species, it is very important to determine their concentration level in the frying oil, because the latter penetrates fried foods, thus favoring their accumulation therein which could cause negative health effects. Different rapid tests are commercially available today to monitor frying oil, which are simple, quick, cheap, and do not require specialized personnel to perform them. Their operation principle is based on measurements of viscosity, dielectric constant, and fatty acid content [167].

Interdigitated electrodes, as capacitive sensors, have been used for the assessment of frying oil quality, considering the relation between electrical capacitance and the content of oil-degradation products [168,169,170,171]. Clearly, this method can be applied only to low-polarity samples, like edible frying oils where the buildup of polar molecules can effectively increase the dielectric constant.

## 5. Overview of Biosensors Applications

**Figure 4:**
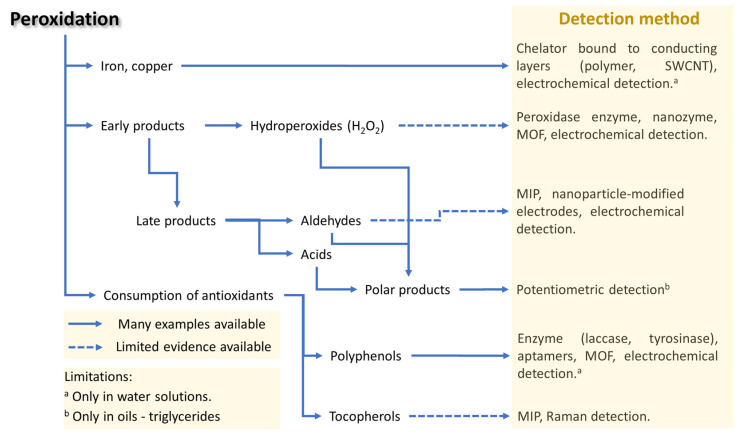
Summary of the application of biosensors to address peroxidation. SWCNT = single-walled carbon nanotubes; MOFs = metal–organic frameworks; MIP = molecularly imprinted polymers.

Figure 4 reports the main molecules involved in the various steps of peroxidation, encompassing the initiation species (metals), oxidation products (classified depending on the formation stage), and antioxidants.

The analytes are connected by arrows to the corresponding biosensor technology, wherein solid and dotted lines represent, respectively, convincing or somewhat weak evidence in this specific sector, mainly ascribable to the low number of examples reported in the literature. From this figure, it can be observed that metals, polyphenols, and polar compounds can potentially be detected in complex systems like food using the existing technology, whereas organic hydroperoxides, aldehydes, and tocopherols still require further studies.

### Examples of Applications in Food: Milk and Plant-Based Milk Alternatives

Oxidative stability is a major concern for extending shelf-life and the long-time conservation of milk and plant-based milk alternatives (obtained from soybean, almond, rice, oats, hazelnut, coconut, etc.) [172]. This issue is more severe for preparations with higher concentrations of PUFA, which can be added on purpose (such as omega-3 fatty acid fortification) or occur naturally [173].

The precise processes causing the oxidation of the various products can vary due to their differing compositions. For instance, the naturally occurring enzyme lactoperoxidase can cause protein oxidation in milk while also helping to regulate bacterial growth [174]. Similarly, when milk is exposed to light, the native component riboflavin acts as a photosensitizer, thus promoting oxidation [175]. Conversely, the elevated PUFA concentration in some plant-based milk substitutes, like soybean beverages, promotes major lipid peroxidation when particular stimuli (like traces of iron) are present [175]; moreover, oxidation can specifically lead to a decrease in the polyphenolic content of plant-based milk alternatives. Another relevant aspect to consider is the interaction of lipids and proteins, which can favor the co-oxidation of the macromolecules [176]; this phenomenon has been observed in milk [177].

The oxidation biomarkers are universal and indicate the formation of radicals, primary oxidation products (H_2_O_2_ and hydroperoxides), and secondary oxidation products (aldehydes, protein carbonyls), regardless of the cause and specific oxidative pathway. The oxidation markers that may be considered for milk and plant-based milk alternatives are peroxides [173], malondialdehyde [172], volatile aldehydes (hexanal, 2-hexenal, heptanal, 2-heptenal, octanal, 2-octenal, nonanal, etc.) [173,175], protein carbonyls [174,175], polyphenols [172], and dimethyl sulfide [175].

In order to be useful for monitoring the stability of complex food matrices, a biosensor for peroxidation often has to overcome problems caused by a lack of selectivity and/or low sensitivity. For example, in milk or plant-based milk alternatives, there are reducing sugars that may interfere with sensing of aldehydes formed by peroxidation. Similarly, peroxides may be confounded by H_2_O_2_ produced by lactoperoxidase. Moreover, the detection of primary and secondary products of peroxidation should be effective for low analyte concentrations (a few parts per million), because at larger concentrations rancid off-flavors start to develop. To assess consumer acceptance of dairy products based on appearance and flavor, the sensory analysis remains the primary method. In fact, trained sensory panels can detect off-flavors like rancidity in milk and milk powders. The International Dairy Federation has reported that the sensory threshold for detecting a rancid flavor in milk is typically between 1.5 and 2.0 mEq of FFA/100 g fat. Values exceeding approximately 1.5 mmol of FFA L^−1^ are considered to reach an unacceptable rancidity threshold. Specific thresholds for individual fatty acids associated with rancidity have also been identified. For caproic acid (C10:0) and lauric acid (C12:0), the detection thresholds are approximately 7 ppm and 8 ppm, respectively [178,179,180].

The combined used of biosensors and sensory analysis to identify and quantify specific VOCs associated with lipid oxidation can provide deeper insights into the oxidation process and help in maximizing the shelf-life of food products without compromising their quality [179].

## 6. Concluding Remarks

The current high interest in biosensors is due to the fact that they provide rapid identification of substances, making them invaluable in urgent situations where quick results are essential. In food quality control, fast detection ensures prompt decisions regarding product safety and shelf-life. Their relatively inexpensive production makes biosensors a cost-effective solution for various applications, promoting widespread adoption in the food industry. Additionally, their miniaturization enables integration into portable devices, facilitating on-site testing and reducing reliance on centralized laboratories.

However, applications to measure lipid peroxidation and antioxidant protection in food require significant work to overcome a number of challenges, the most important of which are selectivity, sensitivity, and the ability to perform the measurement in complex lipid-containing matrices.

The selectivity problem is based on the similitude of the molecules specifically released by lipid peroxidation to other unrelated molecules. For example, peroxidation releases lipid aldehydes and short-chain fatty acids, but the aldehyde functional group can be present in reducing sugars, while fatty acids can also be produced by bacteria. Therefore, biosensors should be able to detect oxidation products having a specific structure, and simply being able to interact with a target functional group may not be sufficient to reach the desired outcome.

Because of human olfaction’s extreme ability to detect peroxidation-related chemicals associated with food spoilage, sensitivity is a major issue in food applications. To be useful in this field, biosensors must be capable of dealing with analyte concentrations in the ppm range.

Working with lipid-containing matrices or bulk lipids appears to be an unresolved issue. For instance, phenolic antioxidants are typically detected using water-based solvents that support enzyme activity and electrochemical methods, whereas lipid-soluble phenols are rarely considered. Another example is that water-soluble hydroperoxides, such as H_2_O_2_, can be easily detected in aqueous media (see Section *Examples of Applications in Food: Milk and Plant-Based Milk Alternatives*), whereas lipid hydroperoxides, which are relevant to food rancidity, are difficult to detect using current biosensor technology.

Despite the challenges, the development of biosensors for lipid peroxidation and antioxidant effect measurement in food is a very promising research field with enormous benefits for food security, safety, and waste reduction, with a relevant potential impact on the improvement of food analysis sustainability.

## Figures and Tables

**Table 1:** Metal–organic framework (MOF) nanocomposites used in electrochemical sensors.

Sample Matrix	Nanomaterial	Target Analyte	Detection Method	LOD	Ref.
Buckwheat tea and orange	Ce-PEDOT nanocomposite	Rutin	ECD	14.7 nM	[103]
Plant extract	glycosyl-MOF-5 and polyacrylonitrile	Quercetin	ECD	83.3 nM	[104]
Human serum	C_3_N_4_-SWCNT/rGO/cyclodextrin MOF	Baicalin	ECD	0.46 nM	[105]
Green tea and urine	Mesoporous carbon and Zr-MOF-801	Gallic acid and luteolin	ECD	0.15 μM	[106]
Pharmaceuticals	Nanocomposite of MIL-100(Fe)-MWCNTs/PEDOT	Kaempferol	ECD	13.2 nM	[108]
Broccoli	Nanocomposite β-CD/C60-GO/Ni-MOF	Kaempferol	ECD	58 nM	[107]
Tea beverage	MOF-doped Au/PEDOT	Catechin/sunset yellow	ECD, DPV	10 nM/150 nM	[110]
Model system (water solution)	Zn CNs/MWCNTs-COOH nanocomposite	Catechin	ECD	10 nM	[109]
Caffeinated drinks and wine	CuO -Cu-MOF	Caffeic acid	ECD	40 nM	[112]
Wine	Mesoporous graphite oxide/Al_2_O_3_, MIT-100(Al)	Caffeic acid	ECD	4 nM	[111]
Pharmaceuticals, wastewater, well water, tap water, tea samples, and biological fluids	ZnTe NRs and Cu MOF	Catechol	ECD	16 nM	[113]
Human serum and urine	Bimetallic MOF-818 and RGO/MWCNTs	Caffeic acid, chlorogenic acid, and gallic acid	ECD, DPV	5.2 nM 5.7 nM 180 nM	[114]
Human serum and urine	Ni-MOF/T-PEDOT	Gallic acid and tinidazole	ECD		[115]
Orange juices	Magnetic nanoparticle Fe_3_O_4_-ZIF-4	Coumaric acid	ECD	180 nM	[116]
Healthcare tablets	Cu HKUST-1	Ascorbic acid	ECD	3 μM	[117]

Abbreviations used in the table: Ce-PEDOT—Ce-doped poly(3,4-ethylenedioxythiophene); ECD—electrochemical detection; MOF-5—functionalized Metal Organic Framework-5; C_3_N_4_-SWCNTs—graphitized carbon-nitride–single-walled carbon nanotubes; Zr-MOF-801—zirconium fumarate metal–organic framework; MIT—mesoporous iron trimesate; MWCNT—multi-walled carbon nanotubes; PEDOT—poly (3,4-ethylenedioxythiophene); β-CD—beta cyclodextrine; C_60_-GO—fullerene graphene oxide; Ni-MOF—nickel metal–organic framework; MOF—metal–organic framework; Au-PEDOT—gold poly (3,4-ethylenedioxythiophene); DPV—differential pulse voltammetry; Zn-CN—zinc-doped carbon nanosheets; ZnTe-NRs—ZnTe nanorods; rGO/MWCNT—reduced graphene oxide/multi-walled carbon nanotubes; Ni-MOF—nickel-based metal–organic framework; T-PEDOT—tubular poly(3,4-ethylenedioxythiophene; ZIF-4—zeolitic imidalozate framework.

**Table 2:** Electrochemical sensors based on nanozyme/MOF sensors for hydrogen peroxide detection.

Sample Matrix	Nanomaterial	Detection Method	LOD	Ref.
Milk samples	BiFeO_3_ and cress peroxidase	AD	70 nM	[124]
Milk samples	Soybean peroxidase/OIH	CV	190 nM	[125]
Real serum, milk, and orange juice	Ag NPs/FeS_2_/ITO nanozyme	ECD	600 nM	[126]
	2D cobalt-MOF nanosheets (nanozyme)	ECD		[129]
Serum samples	2D NiCoM nanosheets	ECD	2.1 μM	[130]
	2D MOF [Co_3_(HOB)_2_]_n_ films	ECD	3.08 nM	[132]
	AgNPs/2D Zn-MOFs	ECD	1.7 μM	[133]

Abbreviations used in the table: AD—amperometric detection; OIH—organic–inorganic hybrid; SBP—soybean peroxidase; CV—cyclic voltammetry; AgNPs—silver nanoparticles; ECD—electrochemical detection; ITO—indium tin oxide; 2D—two-dimensional; HOB—1,2,3,4,5,6-benzenehexaol; NiCoM—trimetallic M (Cu, Fe, Zn).

**Table 3:** Electrochemical sensors for HMF determination based on nanomaterials’ electrode surface modification.

Sample Matrix	Nanomaterial	Detection Method	LOD	Ref.
Beverages	MIP	SWV	50 μM	[135]
Coffee	Electropolymerized MIP	DPV	0.37 mg L^−1^	[136]
Honey samples	NiONPs-modified SPCE	SWV	0.24 ppm	[137]
Food	Cu-Ni bimetallic microparticles	ChAD	3.5 μM	[138]
Honey	NiO-CB (nickel oxide and carbon black)	SWV	5.4 mg kg^−1^	[139]
Molasses	Ag@CMC/GCE	CV and LSV	–	[140]
Honey	Silver microdendrite/SPCE	ECD	1.0 ppm	[141]
Model system (water solution)	Co-based layered double hydroxides	ChAD	0.1 mM	[142]
Coffee	Graphene quantum dots NiAl_2_O_4_—MIP	ECD, SWV	0.30 ng L^−1^	[143]

Abbreviation used in the table: SWV—square-wave voltammetry; ECD—electrochemical detection; DPV—differential pulse voltammetry; ChAD—chronoamperometric detection; CV—cyclic voltammetry; LSV—linear scan voltammetry; MIP—molecularly imprinted polymer; SPCE—screen-printed carbon paste electrode; NiONPs—nickel oxide nanoparticles; NiOCB—nickel oxide and carbon black; Ag@CMC—Ag incorporated into carboxymethyl cellulose; GCE—glassy carbon electrode; GrQD—graphene quantum dots; μP—microparticles; μD—microdendrite; SWV—square-wave voltammetry.

**Table 4:** Developed electrochemical sensors for the detection of heavy metals in different samples.

Sample Matrix	Nanomaterial	Target Analyte	Detection Method	LOD	Ref.
Rice and tap water	MWCNT/carboxamide ligand PIGE	Pb(II) and Cd(II)	ECD, SWV	2.7 nM and 0.92 nM	[149]
Milk	eRGO/Bi	Pb(II) and Cd(II	SWASV	0.8 μg L^−1^ 0.5 μg L^−1^	[150]
Water	GE-cyclam	Cu(II)	AdASV	1.1 nM	[151]
River water, soil, and vegetable (spinach)	Graphene aerogel (GA)-MOF	Cd^2+^, Pb^2+^, Cu^2+^, and Hg^2+^	DPSV	20 nM Cd^2+^, 1.5 nM Pb^2+^, 7 nM Cu^2+^, 2 nM Hg^2+^	[152]
Water samples	Nanomaterial/ionophore	Pb^2+^	ASV	1.0 nM	[153]
Water samples	Nano-sized Pb^2+^ MIP	Pb^2+^	SV	0.6 nM	[154]
Water samples	PDMcT-MWCNTs (poly(2,5-dimercapto-1,3,4-thiadiazole))	Pb^2+^ and Cd^2+^	ASDPV	0.05 μg L^−1^ 0.03 μg L^−1^	[155]
Water samples	Ln-MOF/GCE	Pb^2+^ and Cd^2+^	SWASV	1.10 nM 1.66 nM	[156]
Water samples	GO on ssDNA-PBNP Prussian blue nanoparticles	As	ECD	0.058 ppb	[157]
Water samples	Au/Fe_3_O_4_ nanoparticles	As	ECD, SWASV	0.0215 ppb	[158]
Water samples	SPGE/AuNP	Pb^2+^ and Cu^2+^	SWASV	2.2 ppb 1.6 ppb	[159]
Livestock feed and manure	DNAzyme-SWCNT	Cu^2+^	Resistance	0.65 nM	[160]
Artisanal sugarcane spirit	GE/poly(CA) caffeic acid (CA)-modified graphite electrodes	Pb^2+^ and Cu^2+^	SWASV	3.01 μg/L Pb(II), and 4.50 μg/L Cu(II)	[161]
Water samples	Zn(OH)_2_@CGE	Cu^2+^	SWASV	0.9 nM	[162]
Fruit juice beverages	Hg/CMWCNTs@ZIF-8 -GCE	Zn^2+^ and Cu^2+^	Voltammetry	5.23 × 10^−3^ and 6.52 × 10^−3^ mg L^−1^	[163]

Abbreviation used in the table: MWCNT—multi-walled carbon nanotubes; SWASV—square-wave anodic stripping voltammetry; PIGE—paraffin-impregnated graphite electrodes; eRGO—electrochemically reduced graphene oxide; GA—graphene aerogel; PDMcT—poly(2,5-dimercapto-1,3,4-thiadiazole); GCE—glassy carbon electrode; GO—graphene oxide; PBNP—Prussian blue nanoparticles; SPGE—screen-printed gold electrode; SWCNT—single-walled carbon nanotubes; GE-cyclam—graphite electrode—(1,4,8,11-tetraazamacrocycle); CA—caffeic acid; CGE—carbon graphite electrode; CMWCNT@ZIF-8—carboxylated multi-walled carbon nanotube–zeolitic imidazolate framework; AdASV—adsorptive anodic stripping voltammetry; ASV—anodic stripping voltammetry; SV—stripping voltammetry.
